# Evidence for calcium-mediated perception of plant symbiotic signals in aequorin-expressing *Mesorhizobium loti*

**DOI:** 10.1186/1471-2180-9-206

**Published:** 2009-09-23

**Authors:** Roberto Moscatiello, Sara Alberghini, Andrea Squartini, Paola Mariani, Lorella Navazio

**Affiliations:** 1 Dipartimento di Biologia, Università di Padova, Via U. Bassi 58/B, 35131 Padova, Italy; 2 Dipartimento di Biotecnologie Agrarie, Università di Padova, Viale dell'Università 16, 35020 Legnaro, Padova, Italy

## Abstract

**Background:**

During the interaction between rhizobia and leguminous plants the two partners engage in a molecular conversation that leads to reciprocal recognition and ensures the beginning of a successful symbiotic integration. In host plants, intracellular Ca^2+ ^changes are an integral part of the signalling mechanism. In rhizobia it is not yet known whether Ca^2+ ^can act as a transducer of symbiotic signals.

**Results:**

A plasmid encoding the bioluminescent Ca^2+ ^probe aequorin was introduced into *Mesorhizobium loti *USDA 3147^T ^strain to investigate whether a Ca^2+ ^response is activated in rhizobia upon perception of plant root exudates. We find that *M. loti *cells respond to environmental and symbiotic cues through transient elevations in intracellular free Ca^2+ ^concentration. Only root exudates from the homologous host *Lotus japonicus *induce Ca^2+ ^signalling and downstream activation of nodulation genes. The extracellular Ca^2+ ^chelator EGTA inhibits both transient intracellular Ca^2+ ^increase and inducible *nod *gene expression, while not affecting the expression of other genes, either constitutively expressed or inducible.

**Conclusion:**

These findings indicate a newly described early event in the molecular dialogue between plants and rhizobia and highlight the use of aequorin-expressing bacterial strains as a promising novel approach for research in legume symbiosis.

## Background

Rhizobia are Gram-negative soil bacteria which can engage in a mutualistic association with leguminous plants. Under nitrogen-limiting conditions, rhizobia colonize plant roots and highly specialized plant organs, the nodules, are generated *de novo *on host roots (for a recent review see [1]). When living symbiotically, rhizobia are able to fix atmospheric nitrogen into forms usable by the plant. In return, they receive dicarboxylic acids as a carbon and energy source for their metabolism. Nitrogen is the most frequent limiting macronutrient in many soils, and it is generally supplied as fertilizer. The rhizobium-legume mutualistic association can reduce or eliminate nitrogen fertilizer requirements, resulting also in a benefit to the environment [2].

A successful symbiosis is the result of an elaborate developmental program, regulated by the exchange of molecular signals between the two partners [3]. During growth in the rhizosphere of the host plant, rhizobia sense compounds secreted by the host root and respond by inducing bacterial nodulation (*nod*) genes which are required for the synthesis of rhizobial signal molecules of lipo-chitooligosaccharide nature, the Nod factors. In the host plant, the generation of intracellular Ca^2+ ^oscillations triggered by Nod factors has been firmly established as one of the earliest crucial events in symbiosis signalling; these oscillations are transduced into downstream physiological and developmental responses [1]. It is not known whether there is a parallel key role for Ca^2+ ^in rhizobia.

As in eukaryotic cells, Ca^2+ ^is postulated to play essential functions in the regulation of a number of cellular processes in bacteria, including the cell cycle, differentiation, chemotaxis and pathogenicity [4,5]. Homeostatic machinery that is able to regulate intracellular free Ca^2+ ^concentration ([Ca^2+^]_i_) tightly is a prerequisite for a Ca^2+^-based signalling system, and is known to be present in bacteria [6]. Ca^2+ ^transport systems have been demonstrated in bacteria, with the identification of primary pumps and secondary exchangers, as well as putative Ca^2+^-permeable channels [5,7]. Other Ca^2+ ^regulatory components such as Ca^2+^-binding proteins, including several EF-hand proteins, have been detected and have been putatively identified from genomic sequences [8,9].

In order to establish precisely when and how Ca^2+ ^regulates processes in bacteria it is essential to measure [Ca^2+^]_i _and its changes in live cells. This has proven difficult because of problems in loading fluorescent Ca^2+ ^indicator dyes, such as fura-2, into bacterial cells. However, the recombinant expression of the Ca^2+^-sensitive photoprotein aequorin, which has been demonstrated to be a suitable method to monitor [Ca^2+^]_i _changes accurately in eukaryotes [10-12], has been successfully applied also to bacteria. Challenge of *E*.*coli *[13-17] and the cyanobacterium *Anabaena *sp. PCC7120 [18-21] expressing aequorin with different stimuli resulted in the induction of transient variations of [Ca^2+ ^]_i _with specific Ca^2+ ^signatures.

Here we report the introduction of a plasmid encoding apoaequorin in *Mesorhizobium loti*, the specific symbiont of the model legume *Lotus japonicus*, and the use of this reporter to examine the Ca^2+ ^response of rhizobia to abiotic and biotic stimuli. The results obtained highlight the occurrence in *M. loti *of Ca^2+^-based mechanisms for sensing and responding to cues originating in the rhizosphere.

## Results

### Construction of an inducible reporter system for Ca^2+ ^measurements in rhizobia

The apoaequorin gene was cloned in the broad host-range expression vector pDB1 [22] under the control of the strong synthetic promoter P_syn_, regulated by the lacI^q ^repressor (see Additional file 1). The pAEQ80 plasmid was mobilized by conjugation into the type strain of *M. loti *(USDA 3147^T^).

### Validation of the experimental system

The functioning in *M. loti *of the pAEQ80 plasmid containing the apoaequorin gene was verified by evaluating the level of aequorin expression in an *in vitro *reconstitution assay. Light emitted by total soluble protein contained in the lysates from wild-type and aequorin-expressing *M. loti *cells was monitored after reconstitution of the apoprotein with coelenterazine. The strong luminescence signal detected in protein extracts from *M. loti *cells containing the apoaequorin construct and induced with IPTG confirmed the efficient level of aequorin expression (see Additional file 2).

We analysed whether the introduced pAEQ80 plasmid (10.5 kb) encoding apoaequorin or the expressed protein could affect bacterial cell growth and the symbiotic performance of *M. loti *cells. There is no significant effect on bacterial growth kinetics exerted either by the introduced plasmid or apoaequorin expression. Nodulation efficiency of *M. loti *pAEQ80 cells on the specific plant host *Lotus japonicus *was checked 4 weeks after bacterial inoculation on roots of seedlings grown on nitrogen-free medium. *L. japonicus *roots were found to be effectively nodulated by the transformed bacterial strain, with no differences in nodule number (5 ± 1) and morphological parameters in comparison to seedlings inoculated with wild-type *M. loti*. The presence of bacteria inside nodules was verified by light microscopy (see Additional file 2). Green foliage was indicative of functional symbiosis.

The occurrence in *M. loti *cells of homeostatic control of the internal Ca^2+ ^activity was then verified by preliminary Ca^2+ ^measurement assays in a luminometer after *in vivo *reconstitution of apoaequorin. Unperturbed exponentially growing rhizobial cells showed a steady-state intracellular free Ca^2+ ^concentration ([Ca^2+^]_i_) residing in the submicromolar range (around 500 nM) (see Additional file 2), demonstrating a tight regulation of [Ca^2+^]_i_. No luminescence was detected either in cultures of the non-recombinant strain incubated with coelenterazine or in recombinant cells that had not been exposed to coelenterazine (data not shown), confirming that the recorded signal was due only to Ca^2+^-dependent light emission from aequorin.

### Environmental stimuli are sensed through transient [Ca^2+^]_i _elevations by *M. loti*

**Figure 1:**
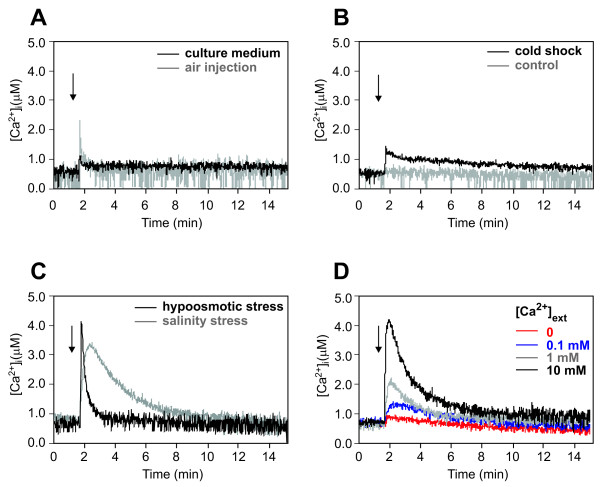
**Ca^2+ ^measurements in *M. loti *cells stimulated with different physico-chemical signals**. Bacteria were challenged (arrow) with: **A**, mechanical perturbation, represented by injection of an equal volume of culture medium (black trace) or 10 volumes of air (grey trace); **B**, cold shock, given by 3 volumes of ice-cold culture medium (black trace); control cells were stimulated with 3 volumes of growth medium kept at room temperature (grey trace); **C**, hypoosmotic stress, given by injection of 3 volumes of distilled water (black trace); salinity stress, represented by 200 mM NaCl (grey trace); **D**, different external Ca^2+ ^concentrations. These and the following traces have been chosen to best represent the average results of at least three independent experiments.

To further validate the experimental system, abiotic stimuli which are known to trigger [Ca^2+^]_i _changes in both plants [23] and cyanobacteria [18,19] were applied to apoaequorin-expressing *M. loti *cells. A mechanical perturbation, simulated by the injection of isoosmotic cell culture medium, resulted in a rapid Ca^2+ ^transient increase (1.08 ± 0.24 μM) that decayed within 30 sec (Fig. 1A). This Ca^2+ ^trace, which is frequently referred to as a "touch response", is often observed after the hand-operated injection of any stimulus [24]. A similar Ca^2+ ^response characterized by an enhanced Ca^2+ ^peak of 2.14 ± 0.46 μM was triggered by a simple injection of air into the cell suspension with a needle (Fig. 1A).

Cold and hypoosmotic shocks, caused by supplying three volumes of ice-cold medium and distilled water, respectively, induced Ca^2+ ^traces with distinct kinetics, e.g. different height of the Ca^2+ ^peak (1.36 ± 0.13 μM and 4.41 ± 0.51 μM, respectively) and rate of dissipation of the Ca^2+ ^signal (Fig. 1B and 1C). As a control, cells were stimulated with three volumes of growth medium at room temperature, (Fig. 1B) resulting in a Ca^2+ ^trace superimposable on that of the touch response (Fig. 1A). These findings eliminate the possible effect of bacterial dilution on changes in Ca^2+ ^homeostasis.

Challenge of *M. loti *with a salinity stress, which has recently been shown to affect symbiosis-related events in *Rhizobium tropici *[25], resulted in a [Ca^2+^]_i _elevation of large amplitude (3.36 ± 0.24 μM) and a specific signature (Fig. 1C).

Variations in the extracellular Ca^2+ ^concentration determined the induction of transient Ca^2+ ^elevations whose magnitude was dependent on the level of external Ca^2+^. After a rapidly induced increase in [Ca^2+^]_i_, the basal Ca^2+ ^level was gradually restored with all the applied external Ca^2+ ^concentrations (Fig. 1D), confirming a tight internal homeostatic Ca^2+ ^control, as previously shown for other bacteria [14,18].

All the above results indicate that aequorin-expressing *M. loti *cells comprise a functionally valid system with which to investigate the involvement of Ca^2+ ^in intracellular transduction of environmental stimuli.

### Host plant root exudates induce in *M. loti *a Ca^2+ ^signal required for activation of nodulation genes

**Figure 2:**
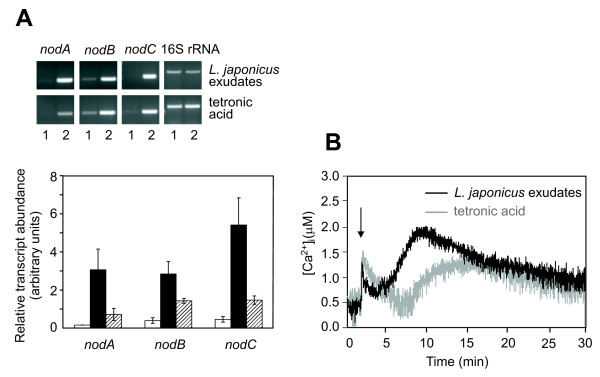
**Effect of plant root exudates and tetronic acid on [Ca^2+^]_i _and *nod *gene expression in *M. loti***. **A**, Analysis of gene expression by semi-quantitative RT-PCR during control conditions (lane 1, white bars) and after 1 h treatment with *L. japonicus *root exudates (lane 2, black bars) or 1.5 mM tetronic acid (lane 2, striped bars). Relative transcript abundance was normalized against 16S rRNA. Data are the means ± SEM of three independent experiments. **B**, Monitoring of [Ca^2+^]_i _changes in *M. loti *cells challenged (arrow) with *L. japonicus *root exudates (black trace) or 1.5 mM tetronic acid (grey trace).

Root exudates from the symbiotically compatible legume *L. japonicus *were collected from 3-week-old seedlings axenically grown in water and applied to *M. loti *cells. The dose used for Ca^2+ ^measurements was in the range that induced significant expression of *nodA*, *nodB*, *nodC *genes in *M. loti *(Fig. 2A). This concentration was found to trigger a transient [Ca^2+^]_i _change characterized by a very rapid increase (1.38 ± 0.23 μM Ca^2+^) followed by a second sustained major Ca^2+ ^peak (2.01 ± 0.24 μM) at about 10 min (Fig. 2B), with a slow decay within the considered time interval (30 min). The observed induction of transient [Ca^2+^]_i _changes in *M. loti *cells suggests a Ca^2+^-mediated perception of signalling molecules contained in host plant root exudates.

Flavonoids are components of root exudates that play a prominent role as inducers of structural *nod *genes in rhizobia. Although flavonoids have been detected in *L. japonicus *seeds [26], those that specifically activate the expression of *nod *genes in *M. loti *have not yet been identified [27,28]. The most common flavonoids, known as *nod *gene inducers in other rhizobia (10 μM naringenin, luteolin, daidzein, kaempferol, quercetin dehydrate) were not able to trigger transient Ca^2+ ^elevations in *M. loti *(data not shown). Tetronic acid, an aldonic acid previously reported to promote Nod factor biosynthesis in *M. loti *[29], was found to induce a detectable Ca^2+ ^response (Fig. 2B). The kinetics of the Ca^2+ ^trace was similar to that induced by crude root exudates, with a prompt Ca^2+ ^spike (1.36 ± 0.16 μM Ca^2+^) and a subsequent flattened dome (maximal Ca^2+ ^value of 1.29 ± 0.08 μM reached around 15 min after the elicitor application). Notably, this second phase of the Ca^2+ ^transient induced by tetronic acid only partially accounted for the larger Ca^2+ ^increase recorded with the whole *L. japonicus *root exudates (Fig. 2B). Likewise, the level of *nod *gene expression induced by tetronic acid was found to be lower (though significantly different from the control, *P *< 0.05) than that generated by total root exudates (Fig. 2A).

**Figure 3:**
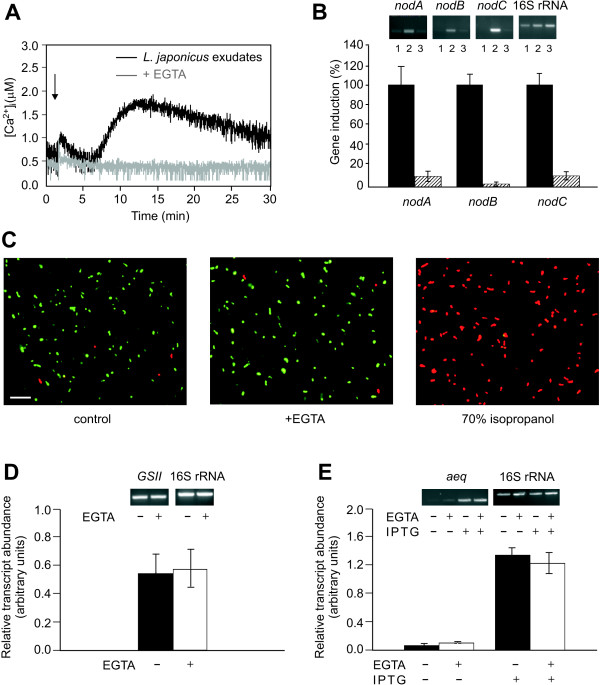
**Effect of EGTA on the Ca^2+ ^response and *nod *gene expression induced by *L. japonicus *exudates**. **A**, *M. loti *cells were treated with *L. japonicus *root exudates (black trace) or pretreated with 5 mM EGTA 10 min before adding *L. japonicus *root exudates (grey trace). **B**, Top: RT-PCR analysis of control cells (lane 1), cells treated for 1 h with *L. japonicus *root exudates (lane 2) and cells pretreated with 5 mM EGTA 10 min before treatment with *L. japonicus *exudates (lane 3). Bottom: Relative percentage of *nod *gene induction in response to *L. japonicus *exudates in *M. loti *cells pretreated (striped bars) or not (black bars) with 5 mM EGTA. Normalization of transcript abundance was done against 16S rRNA. Data are the means ± SEM of three independent experiments. **C**, Viability, monitored with the *Bac*Light Bacterial Viability kit, of *M. loti *cells in control conditions or incubated with 5 mM EGTA for 1 h 10 min. As positive control, cells were treated with 70% isopropanol. Live cells fluoresce green, dead cells fluoresce red. Bar = 10 μm. **D**, Top: RT-PCR analysis of the expression of the housekeeping gene glutamine synthetase II (*GSII*) in *M. loti *cells in the absence (-) or presence (+) of 5 mM EGTA. Bottom: Relative transcript abundance of *GSII *was normalized against 16S rRNA. Bars represent SEM. **E**, Top: RT-PCR analysis of the inducible aequorin (*aeq*) gene in *M. loti *cells in the absence (-) or presence (+) of 5 mM EGTA and 1 mM IPTG. Bottom: Relative transcript abundance of *aeq *was normalized against 16S rRNA. Bars represent SEM.

Pretreatment of rhizobial cells with the extracellular Ca^2+ ^chelator EGTA for 10 min effectively inhibited both the transient Ca^2+ ^elevation (Fig. 3A) and *nod *gene activation (Fig. 3B) induced by *L. japonicus *root exudates. This indicates that the main source of the observed Ca^2+ ^response is the extracellular medium, and that the elevation in [Ca^2+^]_i _is required for *nod *gene induction. Cell viability, monitored by the *Bac*Light Bacterial viability assay, was not altered by incubation with the Ca^2+ ^chelator (Fig. 3C). The expression of both constitutive (glutamine synthetase II and 16S rRNA) and inducible (aequorin) genes was not significantly affected by EGTA treatment (Fig. 3D and 3E), ruling out possible general effects of extracellular Ca^2+ ^chelation on gene induction.

**Figure 4:**
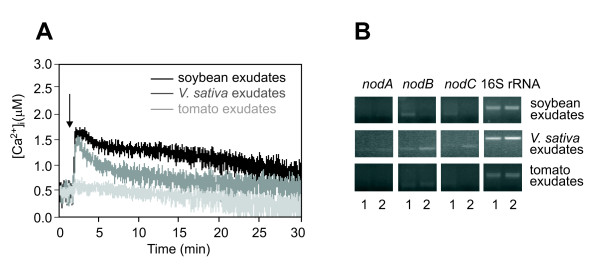
**Monitoring [Ca^2+^]_i _and *nod *gene expression in response to non-host legume and non-legume root exudates**. Bacteria were challenged with root exudates from soybean (**A**, black trace; **B**, lane 2), *V. sativa *subsp. *nigra *(**A**, grey trace; **B**, lane 2) and tomato (**A**, light grey trace; **B**, lane 2). Control cells were treated with cell culture medium only (**B**, lane 1).

To check host specificity of the Ca^2+ ^signal, metabolite mixtures exuded by the non-host legumes soybean and *Vicia sativa *subsp. *nigra *were tested. After an initial rapid and steep Ca^2+ ^rise (1.77 ± 0.34 μM), shared also by the response to *L. japonicus *root exudates, the Ca^2+ ^transients triggered by non-host exudates show very different kinetics, such as a slow rate of decay of the Ca^2+ ^level (Fig. 4A versus Fig. 2B). Pretreatment with EGTA also blocked these transient Ca^2+ ^elevations (data not shown). The distinct Ca^2+ ^signature activated by non-host legumes, together with the lack of activation of *nod *genes (Fig. 4B), suggests the possibility of Ca^2+^-mediated perception by *M. loti *of molecules other than *nod *gene inducers, such as non-specific chemoattractants or other signalling molecules, *e.g. *proteins [30,31] or plant cell wall fragments released during the detachment of border cells from the root tip [32], activating a different Ca^2+ ^signalling pathway. Further confirmation of the specificity of the host plant-induced Ca^2+ ^signalling comes from the complete absence of any detectable Ca^2+ ^change and *nod *gene transcriptional activation by root exudates from a non-legume (tomato) (Fig. 4A and 4B).

## Discussion

Even though Ca^2+^-based signal transduction processes are well-established to underpin plant cell responses to rhizobial informational molecules, a possible involvement of Ca^2+ ^as a messenger in rhizobia in response to plant symbiotic signals has not hitherto been considered. We approached this issue by constructing a *M. loti *strainexpressing the bioluminescent Ca^2+ ^indicator aequorin. The highly sensitive and reliable aequorin-based method is widely used to monitor the dynamic changes of [Ca^2+^]_i _in both eukaryotic [33] and bacterial [18,16] living cells and represents to date the tool of choice for monitoring Ca^2+ ^changes in cell populations [11]. The effectiveness of this recombinant technique has been verified at more than one level, and the results obtained demonstrate the utility of aequorin as a probe to study the early recognition events in rhizobium-legume interactions from the bacterial perspective.

The generation of a well-defined and reproducible Ca^2+ ^transient in *M. loti *cells in response to root exudates of the host plant *L. japonicus *containing *nod *gene inducers is indicative of Ca^2+ ^participation in sensing and transducing diffusible host-specific signals. It cannot be ruled out that the biphasic pattern of the Ca^2+ ^trace (Fig. 2B), monitored by the aequorin method, may be due to an instantaneous synchronized Ca^2+ ^increase in cells immediately after stimulation, followed by a sustained Ca^2+ ^response probably due to the sum of asynchronous oscillations occurring in single cells. Ca^2+ ^oscillations, considered as a universal mode of signalling in eukaryotic cells [34-36] have been proposed to occur in bacteria as well [37].

The significant inhibition of *nod *gene expression obtained when the Ca^2+ ^elevation is blocked indicates that an upstream Ca^2+ ^signal is required for *nod *gene activation. The Ca^2+ ^dependence of *nod *gene expression strongly suggests that the [Ca^2+^]_i _change, evoked by *L. japonicus *exudates, represents an essential prerequisite to convey the plant symbiotic message into rhizobia. All the above results fulfil the criteria required to demonstrate that a Ca^2+ ^transient is a crucial intermediate in a stimulus-response coupling [23] and confirm that Ca^2+ ^signalling is operating in bacteria [5].

The inability of root exudates from non-host legumes and non legumes to duplicate the response induced by *L. japonicus *exudates (encoded in a distinct Ca^2+ ^transient and downstream gene expression) further supports the symbiotic specificity of the host legume-induced Ca^2+ ^signature. The possible relatedness to legume-rhizobium symbiosis of the signals contained in non-host legume exudates is supported by the absence of any Ca^2+ ^response to non-legume exudates. In non-host legume root exudates *M. loti *cells may sense signalling molecules related to the symbiotic process but not strictly specific to the compatible host-microsymbiont pair, which may enable rhizobia to distinguish non-host from compatible plants.

Plant root exudates contain a pool of molecules, both stimulatory and inhibitory, of potential relevance to the molecular signal exchange between the two partners [3]. The use of entire natural mixtures secreted by plant roots represents the first step in the evaluation of rhizobium reactions to plant factors, providing information on the global Ca^2+ ^responses occurring in the bacterial partner early in the symbiosis, even before a physical contact between the two interacting organisms. Further insights into the dynamics of the activated Ca^2+ ^change may come from the comparison with the Ca^2+ ^responses obtained by using fractionated root exudates or purified molecules. This would enable to assess the possible placement of the Ca^2+ ^signal within the NodD-flavonoid gene expression paradigm [38] in different species of rhizobia.

## Conclusion

The above results demonstrate that *M. loti *cells sense host plant symbiotic cues through Ca^2+ ^and indicate that activation of *nod *genes requires an upstream Ca^2+ ^signal. Transgenic rhizobium strains expressing aequorin can be used as a novel approach to the dissection of early events in legume-rhizobium symbiosis, that may shed light on a previously uninvestigated facet - bacterial Ca^2+ ^signalling - of the two-way partner signal exchange and transduction.

## Methods

### Chemicals

Native coelenterazine was purchased from Molecular Probes (Leiden, The Netherlands). Molecular biology reagents were purchased from Promega Co. (Madison, WI, USA), Qiagen (Hilden, Germany) Clontech (Mountain View, CA, USA) and Invitrogen (Paisley, UK). Tetronic acid was obtained from Titolchimica (Rovigo, Italy). Flavonoids (naringenin, luteolin, daidzein, quercetin dehydrate) and all other reagents were obtained from Sigma-Aldrich (St. Louis, MO, USA).

### Bacterial strains and growth conditions

*Mesorhizobium loti *strain USDA 3147^T ^was kindly provided by Peter Van Berkum (USDA, Beltsville MD) and was grown in minimal BIII medium [39] with or without 30 μg/ml kanamycin, as appropriate, at 28°C with shaking (170 rpm). *E. coli *was grown in LB medium at 37°C.

### Cloning of the apoaequorin gene and introduction into *M. loti*

The terms aequorin and apoaequorin refer to the bioluminescent protein with and without, respectively, the prostethic group coelenterazine. The apoaequorin cassette, given by the apoaequorin cDNA fused to the first 27 nucleotides encoding hemoagglutinin (HA1-AEQ) [40] was amplified by PCR with primers designed to obtain a 5' *Xba*I site and to leave out the ATG start codon, already present into the P_syn _promoter of the expression vector pDB1 [22]. The correct translation frame was maintained by adding a nucleotide between the 5' *Xba*I site and the apoaequorin gene. The primers used to obtain the apoaequorin cassette were: 5'-CCTACTCTAGATAAGCTTTATGATGTTCCT-3'and 5'TGATAGCATGCGAATTCATCAGTGTTTTAT-3'. PCR was run with the following parameters: 5 min at 94°C as start step; 30 s at 94°C, 30 s at 58°C, 1 s at 72°C for 30 cycle and 5 s at 72°C as a final step using PLATINUM^® ^Taq DNA polymerase (Invitrogen). To obtain a 3' *Xba*I site, the amplicon was then cloned into the pCR 2.1 plasmid by using TA Cloning^® ^technology (Invitrogen), originating p2.1AEQ. Digestion with *Xba*I of this intermediate plasmid released the HA1-AEQ coding region, which was then ligated into the *Xba*I site of pDB1 under the control of the strong isopropylβ-D-thiogalactoside (IPTG)-inducible synthetic promoter P_syn_. The apoaequorin gene containing construct (pAEQ80, see Additional file 1) was mobilized to *M. loti *3147^T ^from *E. coli *by triparental conjugation using plasmid pRK2013 as helper [41]. Transconjugants were selected on BIII agar containing 50 μg/ml kanamycin.

### Growth kinetics of the recombinant strain

To determine the effect of the plasmid presence and of apoaequorin expression on bacterial cell growth, *M. loti *wild-type or containing pAEQ80 (plus or minus IPTG) were grown in 30 ml of BIII medium (supplemented or not with 30 μg/ml kanamycin, as appropriate) as described above. Growth was determined by monitoring turbidity at 600 nm.

### *In vitro L. japonicus *nodulation tests

*In vitro *nodulation studies were carried out as described by [42]. Briefly, seeds of *L. japonicus *B-129 GIFU were transferred after sterilization on 0.1% Jensen medium solidified with 1% agar. Inoculation with bacterial suspensions of *M. loti *wild-type or containing pAEQ80 (5·10^7 ^cells/root) was carried out 4 days after seed germination. *Lotus *seedlings, before and after infection, were grown at 24°C with 16 h light and 8 h dark. Growth and nodulation pattern were monitored for 4 weeks after inoculation. Microscopy observations were carried out with a Leica MZ16 stereomicroscope equipped with a DFC 480 photocamera. To check the actual occurrence of bacteria inside the nodules, they were squeezed and the content stained with 5 μg/ml 4',6-diamino-2-phenylindole (DAPI). Samples were observed with a Leica DMR fluorescence microscope. Images were acquired with a Leica IM500 digital camera.

### Expression of apoaequorin

A loopful of *M. loti *USDA 3147^T ^pAEQ80 grown on BIII plates was used to inoculate 30 ml of BIII medium supplemented with 30 μg/ml kanamycin and 1 mM IPTG and grown at 28°C overnight, until an absorbance at 600 nm of approximately 0.25 was reached (after about 18 h).

### *In vitro *reconstitution of apoaequorin to aequorin

*M. loti *suspension cultures (300 ml) were grown to mid-exponential phase (A_600 nm _= 0.25), pelletted by centrifugation at 3000 *g *for 10 min at 4°C, washed twice with fresh medium, and finally resuspended in 2 ml reconstitution buffer (Tris-HCl 150 mM, EGTA 4 mM, supplemented with 0.8 mM phenylmethylsulfonyl fluoride, pH 8.0). Bacteria were lysed by 3 cycles (30 s each) of sonication at 35 Hz (Fisher Sonic, Artek Farmingdale, NY, USA), each followed by 30 s on ice. Non lysed bacteria were pelletted and discarded by centrifugation (1600 *g *for 15 min at 4°C). Protein concentration in the supernatant was estimated using the Bio-Rad (Hercules, CA) protein assay according to manufacturer's instructions. Total soluble proteins were resuspended at 1 μg/μl in reconstitution buffer and incubated with 1 mM β-mercaptoethanol and 5 μM coelenterazine for 4 h in the dark at 4°C. Aequorin luminescence was detected from 50 μl of the *in vitro *aequorin reconstitution mixture, containing 25 μg of total soluble protein diluted 1:2 with the same buffer and integrated for a 200 s time interval after the addition of an equal volume of 100 mM CaCl_2_.

### *In vivo *reconstitution of apoaequorin to aequorin

Mid-exponential phase cells (30 ml) were harvested by centrifugation at 2300 *g *for 15 min at room temperature and the cell pellet was washed twice in 5 ml BIII medium with intermediate centrifugation as described above. Cells were then incubated in BIII medium containing 5 μM coelenterazine in the dark for 1 h 30 min under shaking. After two washes as above, cells were resuspended in BIII medium and allowed to recover for 10 min prior to Ca^2+ ^measurement experiments.

### Root exudate production

Seeds of *Lotus japonicus *GIFU ecotype, soybean, *Vicia sativa *subsp. *nigra *and tomato were surface sterilized and allowed to germinate for three days on moistened filter paper at 24°C in the dark. Subsequently, seedlings were transferred aseptically on polystyrene grids covered with nylon meshes in sterile plastic containers containing different volumes of sterile H_2_O, depending on the seed and seedling size (on average 5 ml of H_2_O per seedling). After 3 weeks of germination crude root exudates were collected, filtered and lyophilized. The pellet was resuspended in BIII medium (50 μl per single root exudate) for cell treatments.

### Ca^2+ ^measurements with recombinant aequorin

Aequorin light emission was measured in a purpose-built luminometer. Bacteria (50 μl) were placed, after aequorin reconstitution, in the luminometer chamber in close proximity to a low-noise photomultiplier, with a built-in amplifier discriminator. The output of the discriminator was captured by a THORN-EMI photon counting board (Electron Tubes Limited, Middlesex, UK) and the luminescence data were converted off-line into Ca^2+ ^concentration values by using a computer algorithm based on the Ca^2+ ^response curve of aequorin [40]. All stimuli were administered to cells by using a light-tight syringe through the luminometer port. The experiments were terminated by lysing the cells with 15% ethanol in a Ca^2+^-rich solution (0.5 M CaCl_2 _in H_2_O) to discharge the remaining aequorin pool. For experiments performed in the presence of different external Ca^2+ ^concentrations, cells were extensively washed and resuspended in buffer A (25 mM Hepes, 125 mM NaCl, 1 mM MgCl_2_, pH 7.5), as described by [16]. When needed, cells were pretreated for 10 min with 5 mM EGTA.

### Bacterial cell viability assay

Bacterial cell viability was monitored by the LIVE/DEAD^® ^BacLight™ Bacterial Viability kit (Molecular Probes), according to manufacturer's instructions. This fluorescence-based assay use a mixture of SYTO 9 and propidium iodide stains to distinguish live and dead bacteria. Bacteria with intact cell membranes stain fluorescent green, whereas bacteria with damaged membranes stain fluorescent red. Samples were observed with a Leica 5000B fluorescence microscope. Images were acquired with a Leica 300F digital camera using the Leica Application Suite (LAS) software.

### Semi-quantitative RT-PCR experiments

*M. loti *cells grown to mid-exponential phase and treated as for Ca^2+ ^measurement experiments (see above) were incubated for 1 h with plant root exudates, tetronic acid or cell culture medium only (as control). To stabilize RNA, bacteria were treated with the RNA protect Bacteria Reagent (Qiagen). Bacterial cell wall was then lysed with 1 μg/ml lysozyme (Sigma) in TE buffer. Total RNA was first extracted using RNeasy Mini kit (Qiagen) and, after DNAse I treatment (Promega), quantified. RNA (5 μg) was primed with Random Decamers (Ambion), reverse transcribed with PowerScript Reverse Transcriptase (Clontech) and diluted 1:5. 5 μl of diluted first-strand cDNA were used as a template in a 50 μl PCR reaction solution. Reverse transcription (RT)-PCR was performed with 5 μl diluted first-strand cDNA. The oligonucleotide primers were designed against *nodA*, *nodB*, *nodC *and glutamine synthetase II (*GSII*) sequences from *M. loti *[43] and the aequorin gene (*aeq*) from *Aequorea victoria *[44], using Primer 3 software. To amplify 16S rRNA gene, Y1 and Y2 primers were used [45].

The thermal cycler was programmed with the following parameters: 20 s at 94°C, 30 s at 68°C and Advantage 2 Polymerase mix (Clontech) was used as Taq polymerase. PCR reactions were allowed to proceed for different number of cycles to determine the exponential phase of amplification. Densitometric analysis of ethidium bromide-stained agarose gels (0.5 μg/ml) was performed using QuantityOne software (Bio-Rad). RT-PCR experiments were conducted in triplicate on three independent experiments. The primer sequences used to obtain amplicons were: 5'-TATGAGCCGACCGGAGCCTTTAAT-3' and 5'-CCGTATAGACCGAGTTCAGCGACAA-3' for *nodA*, 5'-ATACTCGATGTGCTGGCGCAAAAT-3' and 5'-GCCTGGTTCGCCTCAAATACTTCAC-3' for *nodB*, 5'-CCACCTTACGATCCTGATGCTGAAA-3' and 5'-CAATATTCTGGCCAATCACGTCCAA-3' for *nodC*, 5'-ACCGAGACTTACGGCATCGACATC-3' and 5'-GCGACGCCATAGCTAAACTTGTTCC-3' for *GSII*, 5'-TAACCTTGGAGCAACACCTGAGCAA-3'

5'-ATACGGATGAGCGTTGGTTCGTTTT-3'for aequorin, Y1 (5'-TGGCTCAGAACGAACGCTGGCGGC-3') and Y2 (5'-CCCACTGCTGCCTCCCGTAGGAGT-3') for 16S rRNA. Amplicons were sequenced by BMR Genomics (Padova, Italy).

## Authors' contributions

RM cloned the apoaequorin gene, carried out the RT-PCR experiments and participated in the Ca^2+ ^measurement experiments. SA and AS introduced the apoaequorin gene into *E. coli *and *M. loti*. LN performed the nodulation studies, prepared the plant root exudates and was involved in acquisition and interpretation of Ca^2+ ^measurement data. MP and LN conceived of the study, designed the experiments and wrote the paper. AS helped with manuscript discussion and participated in its editing. All authors read and approved the final manuscript.

## Supplementary Material

Additional file 1 **Map of the apoaequorin-expressing plasmid pAEQ80**. Abbreviations: P, IPTG-inducible synthetic promoter (P_syn_); HA1-AEQ, cloned apoaequorin cDNA with hemoagglutinin epitope; Km^R^, kanamycin resistance gene; lacI^q^, constitutive lac repressor gene. Relevant restriction endonuclease sites are also shown. Click here for file

Additional file 2 **Validation of the aequorin-expressing *M. loti *experimental system**. **A**, Analysis of aequorin expression in *M. loti *based on an *in vitro *reconstitution assay. Data are the means ± SEM of three experiments. **B**, Effect of pAEQ80 plasmid and expressed recombinant apoaequorin on *M. loti *cell growth. Data are the means of two independent experiments. **C**, Nodulated root of *L. japonicus *4 weeks after inoculation with the recombinant *M. loti *strain. Bar = 2 mm. **D**, DAPI staining of *M. loti *cells USDA 3147^T ^pAEQ80 squeezed from a young nodule. Bar = 10 μm. **E**, Monitoring of intracellular Ca^2+ ^concentration ([Ca^2+^]_i_) in resting *M. loti *cells grown to mid-exponential phase. Click here for file
