# Supplementary figures and images for: Evidence for calcium-mediated perception of plant symbiotic signals in aequorin-expressing Mesorhizobium loti
Source: BMC Microbiol. 2009 Sep 23;9:206. doi: 10.1186/1471-2180-9-206 (PMC2759959; doi:10.1186/1471-2180-9-206)

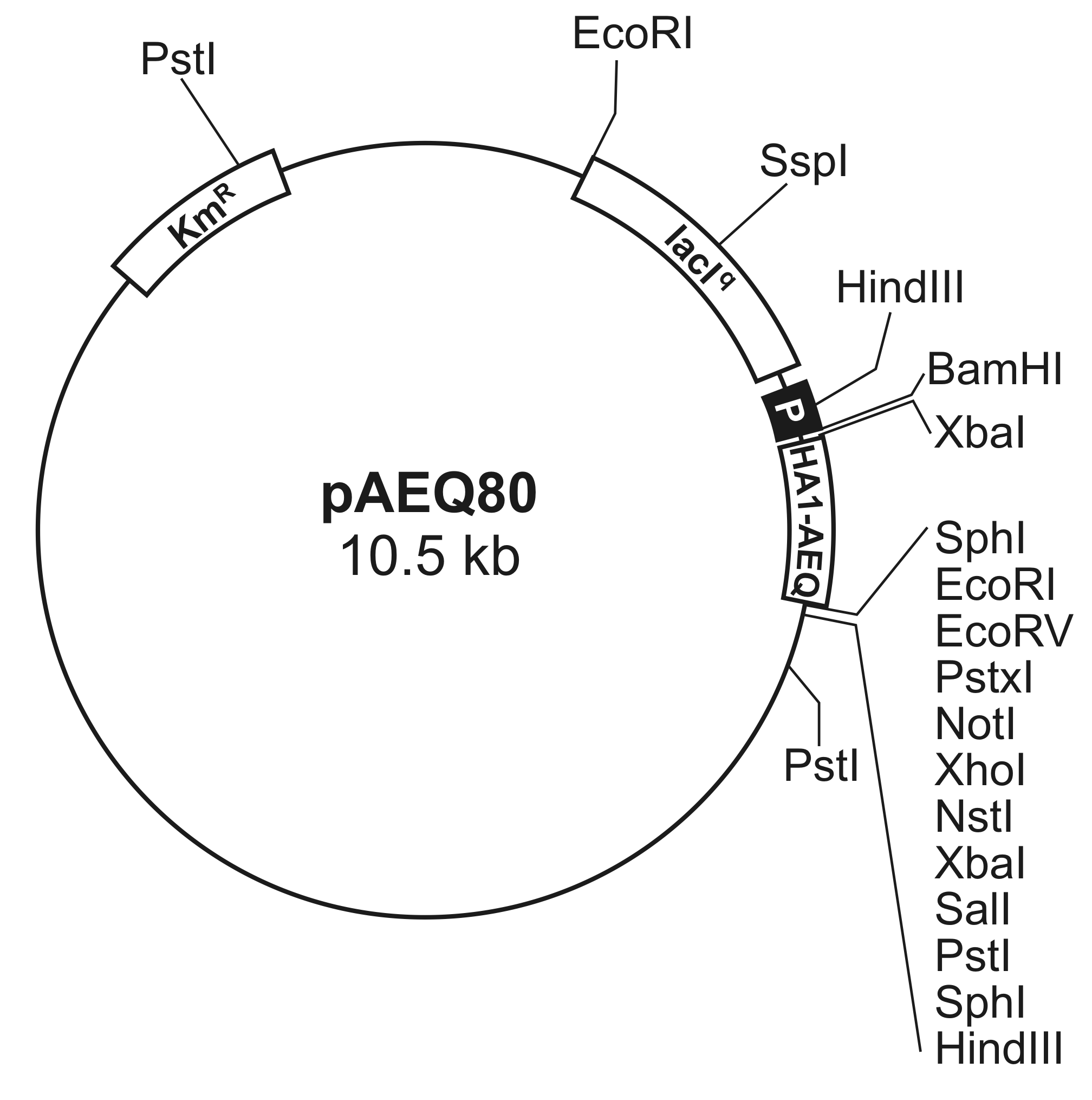

Supplement: Additional file 1 — Map of the apoaequorin-expressing plasmid pAEQ80. Abbreviations: P, IPTG-inducible synthetic promoter (Psyn); HA1-AEQ, cloned apoaequorin cDNA with hemoagglutinin epitope; KmR, kanamycin resistance gene; lacIq, constitutive lac repressor gene. Relevant restriction endonuclease sites are also shown. [file 1471-2180-9-206-S1.tiff]

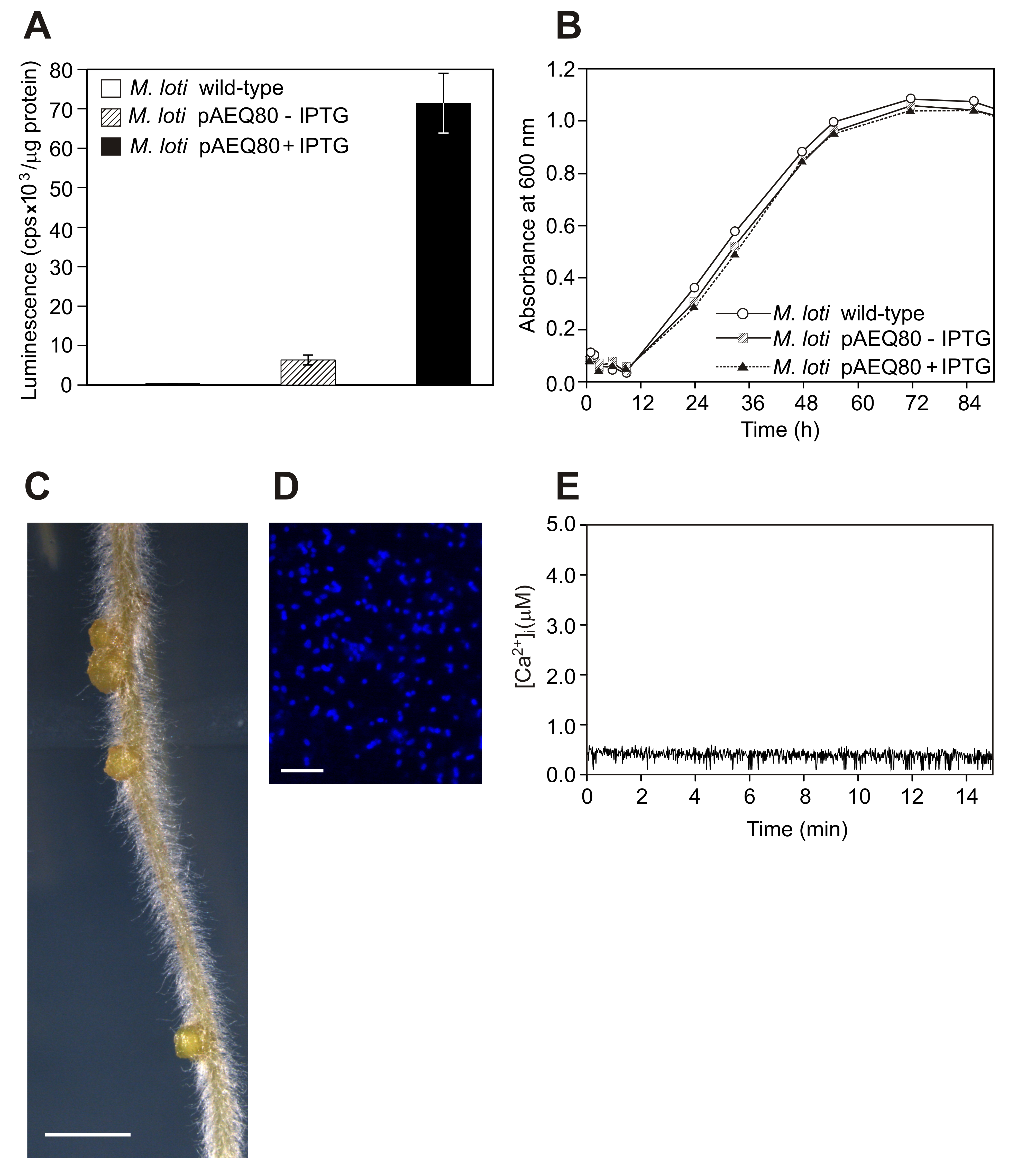

Supplement: Additional file 2 — Validation of the aequorin-expressing M. loti experimental system. A, Analysis of aequorin expression in M. loti based on an in vitro reconstitution assay. Data are the means ± SEM of three experiments. B, Effect of pAEQ80 plasmid and expressed recombinant apoaequorin on M. loti cell growth. Data are the means of two independent experiments. C, Nodulated root of L. japonicus 4 weeks after inoculation with the recombinant M. loti strain. Bar = 2 mm. D, DAPI staining of M. loti cells USDA 3147T pAEQ80 squeezed from a young nodule. Bar = 10 μm. E, Monitoring of intracellular Ca2+ concentration ([Ca2+]i) in resting M. loti cells grown to mid-exponential phase. [file 1471-2180-9-206-S2.tiff]
